# Practicing evidence-based Orthodontics: How to critically appraise a randomized controlled trial

**DOI:** 10.1590/2176-9451.20.2.012-015.ebo

**Published:** 2015

**Authors:** Joana Cunha-Cruz

**Affiliations:** 1 University of Washington, University of Washington, Department of Oral Health Sciences, Seattle, WA, United States, Research assistant professor, University of Washington, Department of Oral Health Sciences, Seattle, WA, United States

An 11-year-old girl visited her general dentist for a routine check up. Upon clinical and radiographic examination, no signs of dental caries were observed, but the dentist noticed that her maxillary canines may have been impacted and referred the patient to the orthodontist. After clinical and radiographic examination, the orthodontist concluded that the teeth were palatally displaced and needed to make a decision for a course of action. The orthodontist suggested extracting the deciduous canines. The parents were hesitant and wanted to wait for the deciduous teeth to exfoliate and the permanent teeth to erupt. The orthodontist then decided to look into published literature for the best evidence available for the treatment of impacted canines. The orthodontist decided to use the five steps of Evidence-based practice: Ask, Acquire, Appraise, Apply and Assess (Table 1). 

**Table 1 -:** The five steps of evidence-based practice.

Stage	Action
ASK a focused question	Use PICOT: Population, Intervention, Comparison, Outcome and Time.
ACQUIRE the best epidemiological evidence to answer the question	Search Pubmed Clinical Queries using keywords and therapy filter with narrow scope and choose the research paper with the highest level of evidence.
APPRAISE evidence for its validity, magnitude of effect and precision	Use RAMBOMAN: Recruitment, Allocation, Maintenance, Blinding or Objective Measurement and Analysis.
APPLY evidence to practice	Use X factor: best available evidence, clinical condition, patient’s preference and values and experience and clinical judgment of the health professional.
ASSESS actual practice against best evidence-based practice	Re-evaluate your practice and evidence.

» Ask a focused question: Use the PICOT (Population, Intervention, Comparison, Outcome, Time) acronym to create a focused question. The clinical question was: In an 11-year-old female patient with suspected palatally displaced maxillary canines, does the extraction of the deciduous canine, compared to no treatment or watchful waiting, result in successful eruption of canines after 4 years? 

» Acquire the evidence: The orthodontist performed a "Narrow scope" search in Pubmed Clinical Queries^1^, category "Therapy", using some of the terms defined in the PICOT question (tooth impaction, canines, extraction). Of the studies retrieved in the search, the orthodontist selected three. 

In the first study, a female patient had deciduous canine extracted and had the permanent canine successfully erupted. In the second study, orthodontists invited their patients, who either would have deciduous canines extracted or did not receive any treatment, to participate in the study. After 4 years, they assessed how many patients in each group had erupted permanent canines. In the third study, patients were randomly allocated by the investigators to have deciduous canines extracted or to have no treatment at all; patients were followed up for 4 years. At the end of the study, they reported how many patients in each group had erupted canines. 

The first study is a case report; the second, a cohort study; and the third, a randomized controlled trial. The case report does not answer the orthodontist's question because it reports a single patient without comparison with a group of patients who did not receive any treatment. The cohort study, however, does have a comparison group, but the decision on whether to extract or not the deciduous canines was based on the clinician's preference and may be biased if the reasons for providing treatment were based on better prognosis, for instance. The orthodontist chose the randomized controlled trial in which assignment to treatment and comparison groups was done by a random process; which is less likely to be biased by personal preferences or favorable prognosis. Then the orthodontist retrieved the full article.

» Appraise the evidence: After reading the full text of the research paper,^2^ the orthodontist appraised the evidence for its validity or risk of bias, magnitude of effect, and precision, using a critical appraisal tool (CAT) for clinical intervention studies based on GATE (Graphic Approach to Epidemiology) frame. The GATE frame was developed by EPIQ (Effective Practice, Informatics and Quality Improvement), a group of academics and practitioners from the University of Auckland, set up to help students and practitioners develop their skills in evidence-based practice.^3^ The PICOT question the study was trying to answer is: In 10-13-year-old Caucasian children with maxillary palatally displaced canine, does the extraction of the primary canine, compared to no extraction, result in successful eruption of the permanent canine after 24 months? Participants were selected from public dental clinics and randomly allocated into extraction of the primary canine (n = 44 teeth) or no extraction (n = 45 teeth) groups. The primary outcome, eruption of the canine (canine emerged through the gingiva), was observed up to 24 months after study onset. However, when the permanent canine showed impairment or no change in its position at the 12-month follow-up examination, combined therapy associating surgical exposure and orthodontic treatment was performed, regardless of the group to which the patient belonged (14 teeth from the extraction group and 27 in the control group). Assessment of the risk of bias using RAMBOMAN (Recruitment, Allocation, Maintenance, Blinding or Objective Outcome and Analysis)^3^ (Table 3) suggests adequate randomized sequence allocation and concealment of the allocation, no losses to follow up and intent-to-treat analysis, but lack of blinding of outcome assessors and failure to report measures of variability. The orthodontist also noticed that the selection criteria was very strict; excluding children with more than 2 mm crowding in the maxilla and children with moderate or severe resorption of adjacent teeth either at the start or during the trial.

**Table 2 -:** Asking a focused question: PICOT.

In an 11-year-old female patient with suspected impacted maxillary canines, does the extraction of the deciduous canine, compared to no treatment or watchful waiting, result in acceptable occlusion after 4 years?	
Participants	11-year-old female patient with suspected impacted maxillary canines
Exposure or intervention	Extraction of the deciduous canine
Comparison	No treatment or watchful waiting
Outcome	Permanent canine erupted
Time	4 years

**Table 3 -:** Appraising the evidence: risk of bias and RAMBOMAN.3

Bias	Description	CAT questions
Recruitment	Systematic differences in the recruitment of participants and baseline characteristics of comparison groups.	Were the study setting and eligible population appropriate?
Allocation	Systematic differences in the allocation of participants to exposure and comparison groups.	Were participants allocated appropriately to groups? If a trial, were they randomized? If randomized, was allocation concealed (i.e. knowledge of group allocated to participants concealed from staff and participants until after allocation was documented)?
Maintenance	Systematic differences in the maintenance of participants in exposure and comparison groups during the study.	Did participants remain in the groups they were initially allocated to? Were completeness of follow-up, compliance, contamination and co-intervention acceptable?
Blinding or Objective Measurement	Systematic differences in outcome assessment.	Were outcome assessors unaware if participants were in exposure or comparison groups (Blinding)? And/or Were outcomes objectively measured (eg., based on biopsies; automated tests, x-rays, validated questionnaires)?
Analysis	Error in the analysis of the study results	Were intention-to-treat analyses done? If exposure and comparison groups were not similar at baseline, was this adjusted for the analyses? Were estimates of intervention effects correctly calculated? Were measures of the amount of random error in estimates of intervention effects correctly calculated?

CAT: Critical appraisal tool.

In addition, the sample size was not calculated for the primary outcome of canine eruption, but for a secondary outcome of positional change of the permanent canine. The orthodontist concluded that the trial had low risk of bias, with generalizability limited to children with similar clinical characteristics as those in the trial (e.g. no moderate to severe crowding in the maxilla). 

The results of the study were: 69% and 39% of permanent canines erupted in the extraction group and in the control group, respectively. Measures of association were not reported in the paper, so the orthodontist calculated them using the GATE calculator (an application designed to accompany the GATE CAT for intervention studies used to calculate measures of association and variability)^3^ (Table 4). The relative risk and 95% confidence interval suggest that permanent canines, in patients whose primary canines were extracted ,were 80% more likely to erupt than those whose primary canines were not extracted (with a variation between 20% and 172%). The NNT (number needed to treat) indicates that three patients need to be treated with the extraction of the primary canine for one permanent canine to erupt (with a variation between two and ten patients). Given the low risk of bias, large confidence intervals and short follow-up period, the orthodontist ranked the evidence using GRADE (Grading of Recommendations Assessment, Development and Evaluation) system^4^ as a moderate level 2 study (in a scale of 1 to 5 in which the lower the level, the better the evidence). Based on the best evidence available, the orthodontist made a weak recommendation for extraction of primary canines. 

**Table 4 -:** Measures of association and variability for the study.

	Event rate (95%CI)		Risk ratio (95% CI)	NNT (95% CI)
	Extraction group	Control group		
Canine eruption	69%	39%	1,8	3
	(54%; 80%)	(26%; 53%)	(1.2; 2.7)	(2; 10)

CI: Confidence Interval, NNT: Number needed to treat.

» Apply the evidence to practice: Before finding the research paper, the orthodontist had carefully conducted a clinical and radiographic examination of the patient and communicated with the patient and her parents to assess their preferences. After critically appraising the best evidence available, the orthodontist needs to assess his clinical experience and personal preferences and communicate again with the patient and his/her parents so as to make a clinical decision. Taking into consideration all the elements of the X factor^3^ (Table 1), the patient underwent extraction of primary canines. 

» Assess actual practice against best evidence-based practice: After treating the patient, the orthodontist considered whether treatment was successful and well accepted by the patient and her parents. The orthodontist also pondered what lessons were learned that could be incorporated into practice so as to make a better decision next time. By re-evaluating his/her own practice, the orthodontist is constantly reshaping the orthodontic practice in light of new research evidence and increased clinical expertise. 

## CONCLUSION

Depending on the research question, specific research designs are more appropriate to answer it. They will have corresponding critical appraisal tools, such as those for intervention studies, diagnostic test accuracy, risk factors and prognostic studies, and systematic reviews. In the hierarchy of evidence-based practice, randomized controlled trials are the highest level of evidence, only below systematic reviews of randomized controlled trials. In the present study, we reviewed the appraisal of a single randomized controlled trial for therapy using the 5 five steps of evidence-based practice and the GATE frame (PICOT, RAMBOMAN and X factor)^3^. Using this straightforward framework, practicing orthodontists can incorporate evidence-based methods into their practice.
